# Complete sequence of the closed circular extrachromosomal element of *Naegleria pringsheimi* De Jonckheere (strain Singh)

**DOI:** 10.1128/mra.00806-23

**Published:** 2024-03-21

**Authors:** Brian T. Nguyen, Nora M. Chapman, Niklas Johnson, Holly A. F. Stessman, Steven Tracy, Kristen M. Drescher

**Affiliations:** 1 Department of Medical Microbiology and Immunology, Creighton University, Omaha, Nebraska, USA; 2 Department of Pathology and Microbiology, University of Nebraska Medical Center, Omaha, Nebraska, USA; 3 Department of Pharmacology and Neuroscience, Creighton University, Omaha, Nebraska, USA; University of Maryland School of Medicine, Baltimore, Maryland, USA

**Keywords:** *Naegleria*, CERE

## Abstract

The DNA encoding the ribosomal RNA in *Naegleria* is encoded on closed circular extrachromosomal ribosomal DNA-containing elements (CERE) in the nucleolus. In this report, we describe the sequence of the CERE of *Naegleria pringsheimi* De Jonckheere (strain Singh).

## ANNOUNCEMENT

*Naegleria* encode all their ribosomal DNA (rDNA) in the nucleolus on closed circular extrachromosomal elements [CERE (1–3)], similar to unrelated species including *Entamoeba histolytica* (4). *Naegleria* can exist as cysts, trophozoites, or flagellates (5). The trophozoites are the best-studied life-cycle stage, as the one human pathogenic *Naegleria*, *Naegleria fowleri*, infects when trophozoites enter the nasal cavity and invade the brain (5). One trophozoite has been estimated to contain approximately ~4,000 copies of the CERE (2). Only one study has examined the CERE origin of replication (ori) in *Naegleria*, with a single ori identified in the large non-ribosomal sequence (NRS) of the CERE (6). The NRS between *Naegleria* species are highly variable despite the conservation of their rDNA cistrons (1, 7–10). *Naegleria pringsheimi* De Jonckheere (Singh) was originally classified as *Naegleria gruberi* but has been reclassified as a unique species (11).

*N. pringsheimi* (Singh; ATCC, Manassas, VA, USA) trophozoites were cultured in modified PYNFH (peptone/yeast extract/nucleic acid/folic acid/heme with 10% fetal calf serum) medium at 25°C. CERE DNA was isolated using the Plasmid Mini Kit per the manufacturer’s instructions (Qiagen, Germantown, PA, USA). Supercoiled CERE was isolated from samples electrophoresed on 0.8% agarose gels using the Monarch DNA Gel Extraction Kit (NEB) per the manufacturer’s instructions and digested with BamHI (NEB). Agarose gel electrophoresis indicated the CERE was approximately 16–17 kbp in size. This band was isolated with the Monarch DNA Gel Extraction Kit. No additional size selection was performed.

The Roy J. Carver Biotechnology Center (University of Illinois–Urbana Champaign) performed the sequencing of unsheared DNA using standard Pacific Biosciences protocols. The Agilent Femto Pulse System was used to determine DNA quality. The library was prepared using the PacBio SMRTbell Express Template Prep Kit 3.0 (Pacific Biosciences), and sequencing was performed on the PacBio Sequel IIe (2.0 chemistry, CCS mode, 30-hour movies).

**Fig 1:**
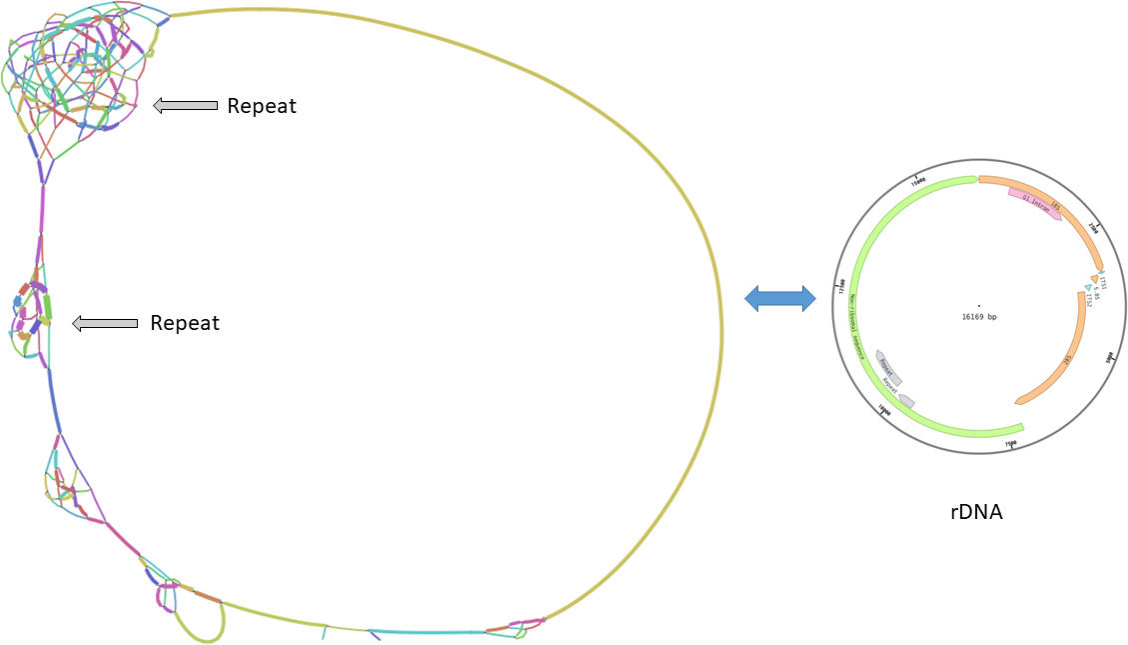
Bandage image of the genome of *N. pringsheimi* as determined using PacBio generated sequences. The location of the rDNA subunits *of N. pringsheimi* is shown in the inset (note that the first nucleotide of the genome is the first nucleotide of the 18S rDNA subunit). The NRS begins at nucleotide 7,187. Repeat regions, characteristic of Naegleria species’ CERE, are indicated by the red arrows.

The *N. gruberi* mitochondrial sequences (GenBank accession no.: NC_002573.1) with at least 65% sequence identity were removed from the 102,563 raw reads (23,737/102,563; 23.1%) (*E*-value 1e-50; remaining settings “default”). The remaining reads (78,826) were separated into FASTA files with reads greater than 5 kbp and less than 17 kbp (11,199/78,826; 14.2% of reads). Reads were assembled using the SPADES Assembler 3.15.5 into contigs and a consensus (options: “—isolate,” “—plasmid—only-assembler”; remaining parameters “default”). CERE characteristics are presented in Table 1. Additional undigested, unsheared CERE DNA was sequenced using Oxford Nanopore Technology (ONT) (plasmidsaurus.com, GridION, V10 Chemistry Library Prep Kit, ligation protocol, R9.4.1 flow cell) to verify the PacBio consensus sequence. No size selection was performed. Bases were called using Guppy (version 6.3.8; SUP). The consensus sequence in GenBank was assembled using PacBio sequences. The ONT sequences were used to resolve repeat sequence regions in the NRS. The overlap of the circular element was identified based on reads with the 5′ and 3′ BamHI digestion site ends. No genome rotation was performed. Figure 1 illustrates the location of the rDNA, NRS, and the repetitive sequences located within the NRS as generated by Bandage (Bioinformatics Application for Navigating *De novo* Assembly Graphs Easily).

**TABLE 1:** CERE characteristics of *N. pringsheimi* De Jonckheere (strain Singh)

Characteristics	Value
Genome size (bp)	16,164
GC content (%)	41.1
Contigs	101 (pre-filtering)^*a*^
Blast alignment to *N. gruberi* rDNA (%)	39
Average read length (bp)	2,624
Total reads (PacBio)	78,826
Total reads (ONT)	1,922
*N*_50_ (PacBio)	8,599
*N*_50_ (ONT)	15,793
Mean read depth (Pac Bio)	5,427.8
GenBank accession	OR045415
SRA accession (raw reads, PacBio)	SRR24044716
SRA accession (raw reads, ONT)	SRR24044730
BioProject	PRJNA950071
BioSample	SAMN33140965

^*a*^ Eighty-five contigs after filtering.

## Data Availability

The consensus sequence has been deposited in GenBank under accession number OR045415.1. The first nucleotide of the 18S rDNA subunit has been designated as position "1." PacBio raw reads have been deposited in the NCBI Sequence Read Archive (SRA) under accession number SRR24044716. Plasmidsaurus (ONT) raw reads are also deposited in the SRA under accession number SRR24044730. The BioProject and BioSample are available as follows, respectively: PRJNA950071 and SAMN33140965. No genome assembly was performed for the ONT reads, as the reads were only used for the verification of the PacBio sequence.
